# Microbial community-level regulation explains soil carbon responses to long-term litter manipulations

**DOI:** 10.1038/s41467-017-01116-z

**Published:** 2017-10-31

**Authors:** Katerina Georgiou, Rose Z. Abramoff, John Harte, William J. Riley, Margaret S. Torn

**Affiliations:** 1 Department of Chemical and Biomolecular Engineering, University of California, Berkeley, CA 94720 USA; 2 Climate and Ecosystem Sciences Division, Lawrence Berkeley National Laboratory, Berkeley, CA 94720 USA; 3 Energy and Resources Group, University of California, Berkeley, CA 94720 USA

## Abstract

Climatic, atmospheric, and land-use changes all have the potential to alter soil microbial activity, mediated by changes in plant inputs. Many microbial models of soil organic carbon (SOC) decomposition have been proposed recently to advance prediction of climate and carbon (C) feedbacks. Most of these models, however, exhibit unrealistic oscillatory behavior and SOC insensitivity to long-term changes in C inputs. Here we diagnose the source of these problems in four archetypal models and propose a density-dependent formulation of microbial turnover, motivated by community-level interactions, that limits population sizes and reduces oscillations. We compare model predictions to 24 long-term C-input field manipulations and identify key benchmarks. The proposed formulation reproduces soil C responses to long-term C-input changes and implies greater SOC storage associated with CO_2_-fertilization-driven increases in C inputs over the coming century compared to recent microbial models. This study provides a simple modification to improve microbial models for inclusion in Earth System Models.

## Abstract (web summary)

Microbial models of soil organic carbon feed into Earth System Models, but many exhibit unrealistic oscillatory behaviour. Here, the authors propose a density-dependent formulation of microbial turnover that improves microbial models, with large implications for global carbon-concentration feedbacks.

## Introduction

Understanding and quantifying the response of soil organic carbon (SOC)—the largest actively cycling terrestrial pool of organic carbon—to climatic and land-use change is imperative for projecting carbon (C) cycle dynamics. In most global- and ecosystem-scale biogeochemical models, SOC decomposition is directly proportional to the size of the soil carbon pool, with additional rate coefficients that account for soil moisture and temperature effects (i.e., “pseudo-first-order models”). This formulation is inherently unable to reproduce potentially critical feedbacks, such as priming (accelerated decomposition) of native SOC stocks due to, for example, increased plant root exudates at elevated CO_2_ concentrations^1, 2^. In fact, it has been widely observed that changes in plant C inputs to soils result in diverse, nonlinear responses of SOC stocks due to microbial population dynamics and competing decomposition and stabilization mechanisms^3–9^. These types of responses are particularly important in the face of atmospheric, climatic, and land-use change, which will affect the amount of plant C inputs that enter the soil—e.g., through changes in plant productivity, rooting depth, allocation, and species distributions^1, 2, 10–12^.

**Fig. 1:**
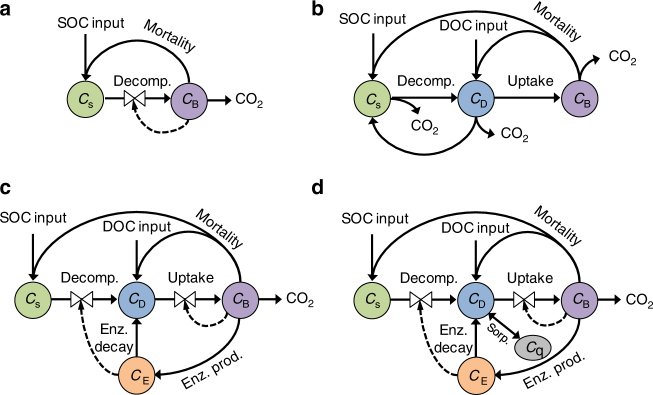
SOC decomposition models compared in this study. **a** Two-pool microbial model with SOC (*C*_S_) and microbial biomass C (MBC; *C*_B_) pools. **b** Three-pool linear, first-order model with SOC, MBC, and dissolved organic C (DOC; *C*_D_) pools. **c** Four-pool microbial model that includes enzymatic (*C*_E_) decomposition of SOC and subsequent assimilation (uptake) of DOC. **d** Five-pool microbial model that includes sorption of organic matter onto mineral surfaces to form mineral-associated organic C (*C*_q_) that is protected from enzymatic attack

Since soil microbial activity mediates SOC decomposition, increases in microbial activity following increases in plant inputs may limit SOC accumulation by stimulating decomposition^3, 6, 13^. Microbial models of SOC decomposition seek to capture this potential carbon-concentration feedback by explicitly representing microbial or enzymatic degradation of SOC^14^. Decomposition rates thus depend not only on the size of the SOC pool but also on the size and composition of the decomposer microbe pool^14–19^. Many microbial models have been proposed in recent years^14, 20–22^, as part of a burgeoning effort to understand and predict soil biogeochemical dynamics. Such models have even been applied to make predictions at the global scale^23, 24^, despite limited mathematical analyses of their dynamics and response to long-term perturbations^25–29^. These models can be grouped according to the complexity they represent (Fig. 1), and require careful theoretical and computational investigation to diagnose emergent dynamics. Within this framework, we used four archetypal models to investigate the observed divergence of model predictions from observations and the emergence of unrealistic, decadal SOC oscillations in response to changes in C input.

Although oscillations of microbial biomass and activity may occur in microsites within the soil in response to a perturbation, such behavior is not commonly observed at the ecosystem scale or at daily and longer timescales^30–32^. That is, oscillations may occur in neighboring soil microsites or within hotspots, but do not appear when aggregated at the field scale because they are out of phase and are smoothed out by destructive interference^33, 34^. However, the latter (larger) scale is that at which these oscillatory models are often applied^23, 27, 35^. There is also a disconnect between the timescales of observed vs. predicted oscillations, where most microbial models exhibit oscillations on the decadal scale^27, 35^. Thus, a key question is one of scale—the oscillations arising from microbial models are not observed at the large temporal and spatial scales at which the models are applied. Microbial models have therefore been avoided in recent studies largely on account of this unrealistic behavior^36^. While several studies have explored the emergence of decadal oscillations^26–28, 35, 37^, a thorough analysis that diagnoses the source of this behavior and proposes modifications to remedy it is still needed.

Many microbial models consider substrate–microbe interactions at the level of individual microbes; however, at the community level, regulatory mechanisms—e.g., competition, space constraints, and other controls that depend on the density of individuals, such as disease and production of toxins—may limit microbial population sizes^34, 38–42^. Indeed, representing such density-dependent processes in models is common in the field of population ecology, where the classic example is the logistic growth model that limits population size to a carrying capacity. While detailed models that include microbial community-level interactions (e.g., microbial competition, functional guilds, and stoichiometric homeostasis) do exist^34, 42, 43^, prominent microbial models of SOC decomposition that are currently applied at regional to global scales do not impose limitations on the size of the microbial population^23, 27, 44^. Thus, microbial biomass in these models can grow indefinitely given a sustained increase in C inputs; whether C inputs double or increase by a factor of ten, the steady-state microbial population will change proportionally. This, consequently, drives SOC back to its pre-disturbance steady state, rendering it insensitive to long-term changes in C inputs. We thus hypothesized that including community-level regulation, via density-dependent microbial turnover, would limit both the unrealistic oscillations and SOC insensitivity to long-term changes in C input currently predicted by such microbial models.

Here we compared linear and microbial SOC models ranging in complexity to systematically diagnose their emergent behavior and attribute unrealistic characteristics to either parametric or structural differences. We incorporated density-dependent microbial turnover to explore the role of community-level regulation on projected SOC feedbacks. Specifically, we evaluated models in their response to long-term changes in plant C inputs by synthesizing observations from 24 long-term (> 5 years) litter manipulations, including the Detritus Input and Removal Treatment (DIRT) and Long-term Bare Fallow (LTBF) experiments, that span a range of soil types. These data are a unique resource for evaluating the models presented in this study and testing our hypothesis and, furthermore, constitute a powerful data set for validating and benchmarking future SOC models. Our findings suggest that density-dependent microbial processes play an essential, but largely overlooked, role in regulating SOC dynamics. We discuss our results in the context of applying SOC models at large spatial scales and make recommendations on model features that are suitable for scaling up to Earth system models.

## Results

### Analytical steady-state solutions of models

For each SOC model (Fig. 1), we derived the analytical steady-state solution as a function of model parameters (Table 1). In the absence of density-dependent microbial turnover (i.e., for density-dependent exponent *β* = 1; see Methods), the steady-state SOC stock (denoted *C*_S_) is not a function of total C inputs (denoted *I*), but only of select system parameters. For example, for the two-pool microbial model with *β* = 1 (Fig. 1a)

1 $${C_{\rm{S}}} = \frac{{{K_{{\rm{M,U}}}} \cdot {k_{\rm{B}}}}}{{\varepsilon \cdot {V_{{\rm{max,U}}}} - {k_{\rm{B}}}}}$$

at steady state (Table 1), where *V*_max,U_ is the maximum microbial assimilation rate, *K*_M,U_ the half-saturation for assimilation, *k*_B_ the microbial turnover rate constant, and *ε* the microbial C-use efficiency (Supplementary Table 1). Conversely, the three-pool linear, first-order model (Fig. 1b) always predicts that the *C*_S_ steady-state solution is directly proportional to total C inputs (Table 1). In the microbial models with *β* = 1 and the three-pool linear model, the steady-state microbial biomass carbon (MBC; denoted *C*_B_) is directly proportional to the C-input rate. For example, for the two-pool microbial model with *β* = 1

2 $${C_{\rm{B}}} = \left( {\frac{{\varepsilon \cdot I}}{{\left( {1 - \varepsilon } \right) \cdot {k_{\rm{B}}}}}} \right).$$

**Table 1:** Steady-state solutions of the SOC decomposition models

Two-pool microbial model (for all *β*)^a^	${{\it{C}}_{\rm{S}}} = \frac{{{{\it{K}}_{{\rm{M,U}}}} \cdot {k_{\rm{B}}} \cdot {{\it{C}}_{\rm{B}}}^{\beta - 1}}}{{\varepsilon \cdot {V_{{\rm{max,U}}}} - {k_{\rm{B}}} \cdot {{\it{C}}_{\rm{B}}}^{\beta - 1}}}$ $,{{\it{C}}_{\rm{B}}} = {\left( {\frac{{\varepsilon \cdot I}}{{\left( {1 - \varepsilon } \right) \cdot {k_{\rm{B}}}}}} \right)^{1/\beta }}$
Three-pool linear model	$${C_{\rm{S}}} = \frac{{f\cdot I + {C_{\rm{D}}}\cdot({f_{\rm{D}}}\cdot{k_{\rm{D}}} + {k_{{\rm{uptake}}}}\cdot{f_{\rm{B}}}\cdot{f_{{\rm{B}} \to {\rm{S}}}})}}{{{k_{\rm{S}}}}},\ $$ $${C_{\rm{D}}} = \frac{{I {\cdot [(1 - f) + f \cdot {f_{\rm{S}}}} ]}}{{\left[ {{k_{{\rm{uptake}}}} + {k_{\rm{D}}} + {k_{{\rm{uptake}}}}\cdot{f_{\rm{B}}}\cdot({f_{{\rm{B}} \to {\rm{S}}}} - 1 - {f_{{\rm{B}} \to {\rm{S}}}}\cdot{f_{\rm{S}}}) - {f_{\rm{D}}}\cdot{k_{\rm{D}}}\cdot{f_{\rm{S}}}} \right]}}, \ $$ ${C_{\rm{B}}} = \frac{{{k_{{\rm{uptake}}}}{C_{\rm{D}}}}}{{{k_{\rm{B}}}}}$
Four-pool microbial model (for all *β*)^a^	${{\it{C}}_{\rm{S}}} = \frac{{{K_{\rm{M}}} \cdot \left( {f \cdot I + {k_{\rm{B}}} \cdot {a_{{\rm{BS}}}} \cdot {C_{\rm{B}}}^\beta } \right)}}{{{V_{{\rm{max}}}} \,\cdot \ \frac{{{r_{\rm{p}}}}}{{{r_{\rm{E}}}}} \,\cdot \,{C_{\rm{B}}} - \left( {f \cdot I + {k_{\rm{B}}} \cdot \,{a_{{\rm{BS}}}} \cdot \,{C_{\rm{B}}}^\beta } \right)}},$ ${C_{\rm{D}}} = \frac{{{K_{{\rm{M}},U}} \cdot \left( {{r_{\rm{p}}} + {k_{\rm{B}}} \cdot {C_{\rm{B}}}^{\beta - 1}} \right)}}{{\varepsilon \cdot {V_{{\rm{max,}}U}} - \left( {{r_{\rm{p}}} + {k_{\rm{B}}} \cdot {C_{\rm{B}}}^{\beta - 1}} \right)}},$ ${k_{\rm{B}}}{C_{\rm{B}}}^{\boldsymbol{\beta }} + {r_{\rm{p}}}{C_{\rm{B}}} = \frac{{\varepsilon \cdot I}}{{\left( {1 - \varepsilon } \right)}}$ (implicit equation for all *β*), ${C_{\rm{E}}} = \frac{{{r_{\rm{p}}} \cdot {C_{\rm{B}}}}}{{{r_{\rm{E}}}}}$
Five-pool microbial model (for all *β*)^a^	*C*_S_, *C*_D_, *C*_B_, *C*_E_ (same as four-pool model for all *β*), ${C_q} = \frac{{{k_{{\rm{ads}}}} \cdot {Q_{\max }} \cdot {C_{\rm{D}}}}}{{{k_{{\rm{des}}}} + {k_{{\rm{ads}}}} \cdot {C_D}}}$

^a^General expressions are given for any value of density-dependent microbial turnover (*β*), where microbial models in the literature do not include density-dependence, i.e., *β* = 1. Parameter definitions can be found in Supplementary Table 1

This implies that for the microbial models with *β* = 1, the ratio of MBC to SOC (*C*_B_/*C*_S_) is proportional to the C-input rate (Supplementary Note 1). With density-dependent microbial turnover (*β* > 1), however, the microbial models predict a sensitivity of the *C*_S_ steady state to C inputs and the *C*_B_ steady state remains responsive, but not proportional, to C inputs (Table 1). This allows the ratio of MBC/SOC to be largely independent of the C-input rate depending on the value of *β*, as is also the case for the three-pool linear model (Table 1). These are fundamental differences among the predictions of microbial models, and between microbial and linear models that are a direct consequence of model structure, and not the parameters.

### Dynamic response of models to perturbations

**Fig. 2:**
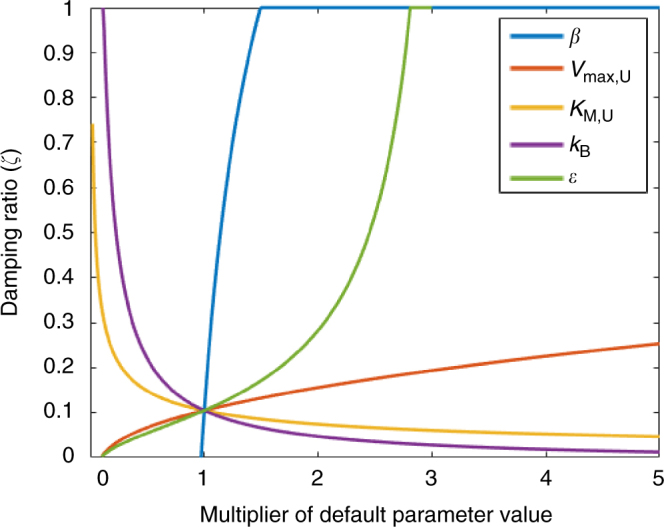
Damping ratio of the two-pool microbial model as a function of the model parameters. The damping ratio (*ζ*) is a metric that depicts the degree of oscillatory behavior of the linearized system near its steady state, where *ζ* = 1 signifies a stable node, 0 < *ζ* < 1 damped (diminishing) oscillations, − 1 < *ζ* < 0 unstable (growing) oscillations, and *ζ* = − 1 an unstable node. Here all parameters were varied independently from their default value (Supplementary Table 1) by the given multiplier (*x*-axis) to illustrate the inherent sensitivity of the oscillations on the parameter values. The model stability is very sensitive to the density-dependence exponent (*β*)

We analytically and numerically explored the stability of each model and its response to C-input perturbations, using a change in both SOC and DOC inputs as a general case. We found that the microbial models in recent literature (i.e., the two-, four-, and five-pool models with *β* = 1; Fig. 1a, c, d) are particularly prone to oscillations as a consequence of their model structure and parameters. For common parameter values (Supplementary Table 1), these models exhibit damped oscillations with a period of 10–20 years in response to an increase in C inputs (Fig. 2; Supplementary Figs. 1–3). This behavior is largely due to an imbalance between the C assimilation term, which has a positive sign and is proportional to *C*_B_ in the differential equation for $\frac{{ {{\rm{d}}{C_{\rm{B}}}} }}{{ {{\rm{d}}t} }}$, and the microbial turnover (mortality) term. Microbial assimilation (hereafter C uptake) is represented as $\varepsilon \left( {\frac{{{V_{{\rm{max,}}U}} \cdot {C_i} \cdot {C_{\rm{B}}}}}{{{K_{{\rm{M,U}}}} + {C_i}}}} \right)$, where *C*_*i*_ represents SOC (*C*_S_) in the two-pool model and DOC (*C*_D_) in the four- and five-pool models. When this C uptake exceeds the mortality term, the microbial population experiences exponential growth until it consumes too much substrate, causing a transient crash in the population until the substrate can recover—and the cycle repeats. We calculated the characteristic damping ratio (*ζ*; see Methods) and the period of oscillation from the eigenvalues of each linearized system and found that they strongly depend on the parameters *V*_max,*U*_, *K*_M__,*U*_, *ε*, and *k*_B_, as well as *β* (Fig. 2; Supplementary Figs. 2, 4 and 5; parameter details in Supplementary Table 1). A damping ratio of *ζ* = 1 (i.e., stable system with no oscillations) is achieved for any *β* ≥ 1.5 in the two-pool model (Fig. 2).

We also analyzed the scenario where the change in C inputs occurs unevenly among pools, entering either the SOC pool (*I*_S_) or the DOC pool (*I*_D_), for example, in contrast to the change in C inputs entering both pools. That is, we performed simulations in which *I*_S_, *I*_D_, or both (*I* = *I*_S_ + *I*_D_) are perturbed (Supplementary Note 2). This analysis was motivated by the potential for the fraction entering each pool (*f*) to change, where *I*_S_ = *f* ⋅ *I* and *I*_D_ = (1 − *f*)  ⋅ *I*. This numerical experiment showed that the transient dynamics and steady-state responses of all models (i.e., the microbial models and the first-order, linear model) depended on which C input (SOC or DOC) was perturbed (Supplementary Figs. 6 and 7). The response of the steady-state SOC stock to a range of C-input treatments (from complete removal to doubling of *I*_S_, *I*_D_, or both) was compared for the three-pool linear model and the four-pool microbial model with and without density-dependent microbial turnover (Supplementary Fig. 7). We observed that without density-dependent microbial turnover, the four-pool model steady-state SOC stock was insensitive to changes in the total C inputs (*I*), consistent with its analytical steady-state solution (Table 1).

### Density-dependent microbial turnover

**Fig. 3:**
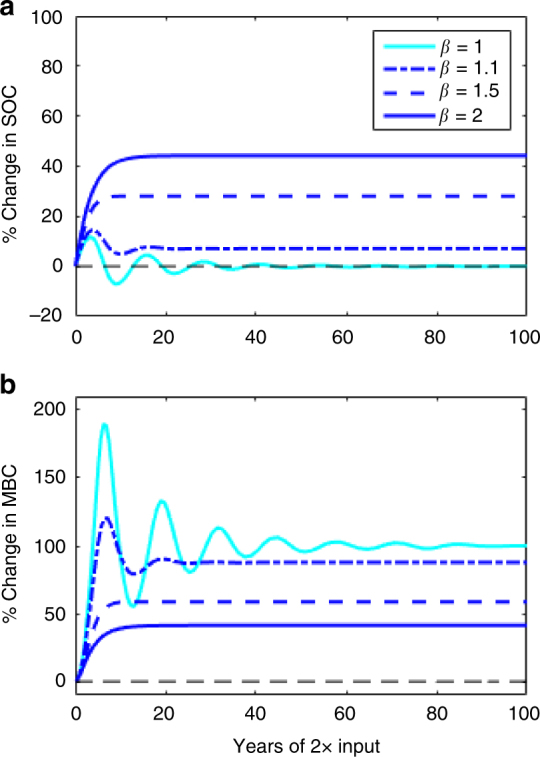
Response of SOC and MBC to a sustained doubling of C inputs in the two-pool microbial model with and without density-dependent microbial turnover. **a** Percent change of modeled SOC following a 2× step increase in total C inputs. **b** Percent change of modeled MBC following a 2× step increase in total C inputs. The value of the parameter *β* depicts the strength of density-dependent microbial turnover, where *β* = 1 corresponds to no density-dependence and *β* = 2 to a strong density-dependence

**Fig. 4:**
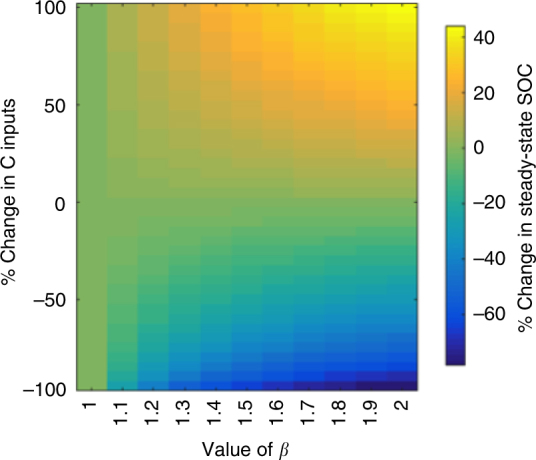
Percent change in the SOC steady state following a range of step changes in C inputs with a range of *β* values in the two-pool microbial model. The value of *β* depicts the strength of density-dependent microbial turnover, where *β* = 1 gives the widely used two-pool microbial model without density-dependence and *β* = 2 gives a strong density-dependence

We evaluated the effects of density-dependent microbial turnover (i.e., setting the mortality rate proportional to (*C*_B_)^*β*^ with *β* > 1) and found that the SOC oscillations in response to changes in C inputs (as observed in the literature for *β* = 1) can be reduced or completely removed (Fig. 3). While an exponent of *β* = 2 leads to logistic growth (see Methods), we note that *β* can take on any value > 1 to reduce SOC oscillations (Figs. 2 and 3). We further explored the effect of *β* on the percent change in steady-state SOC following a range of step changes in total C inputs (Fig. 4). For *β* = 1, the model reduces to the common microbial model where the long-term SOC is insensitive to any change in total C inputs. As the exponent (*β* > 1) increases, however, the long-term SOC becomes increasingly sensitive to changes in C inputs.

In all models without density-dependence, heterotrophic respiration (CO_2_ efflux) is always directly proportional to MBC (Supplementary Figs. 8 and 9). When density-dependence is incorporated, steady-state respiration doubles for a doubling of C inputs, but the steady-state MBC does not double due to constraints that limit population size (Supplementary Figs. 4b, c and 8b, c). This response is effectively an increase in the specific respiration rate as microbial populations increase, since there is proportionally more respiration per unit of MBC.

### Doubling C inputs in models and experiments

**Fig. 5:**
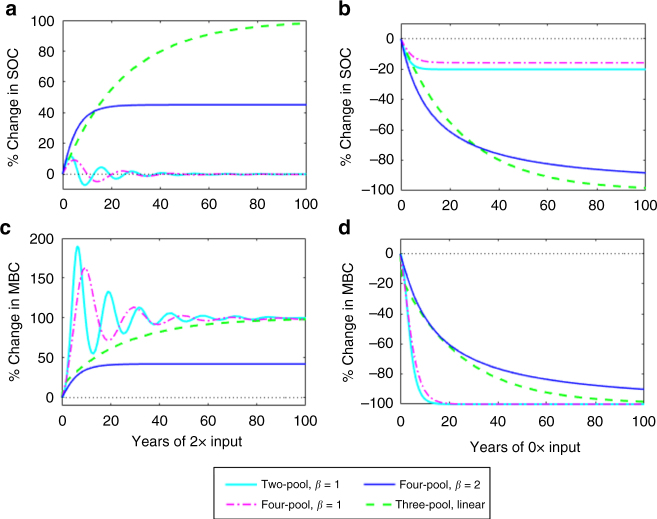
Response of SOC and MBC to doubling and removal of C inputs in models. **a**, **c** Percent change of modeled SOC and MBC following a 2× step increase in inputs. **b**, **d** Percent change of modeled SOC and MBC following sustained 0× inputs. A value of *β* > 1 corresponds to a microbial model with density-dependent microbial turnover. The five-pool microbial model overlaps with the four-pool microbial model for the parameter set in Supplementary Table 1

**Fig. 6:**
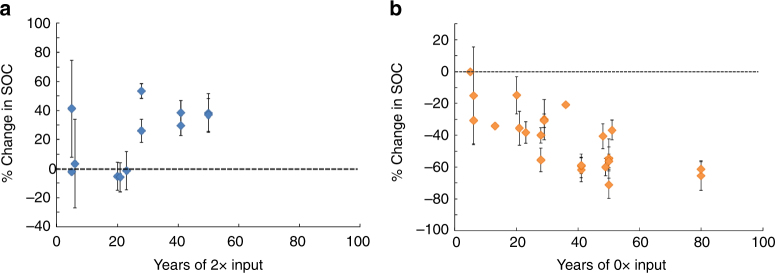
Response of SOC to doubling and removal of C inputs in long-term litter manipulations. **a** Percent change in SOC at Detritus Input and Removal Treatment (DIRT) experiments after a sustained 2× step increase in inputs. **b** Percent change in SOC at DIRT and Long-Term Bare Fallow (LTBF) experiments after sustained 0× inputs. Points indicate means and bars the s.e.m. Individual points are separated by site in Supplementary Fig. 10 and data sources are reported in Supplementary Tables 2 and 3

We compared the response of all four models to a doubling of total C inputs (Fig. 5a, c) with our synthesis of DIRT experiments, in which C inputs to the soil were doubled at six different sites over 5–50 years (Fig. 6a; Supplementary Table 2). The two longest-running DIRT sites, Noe and Wingra Woods (50 years), showed similar increases in SOC stocks in response to doubling C inputs, where SOC approached a new steady state that was ~ 40% higher than the control (Fig. 6a). Similar SOC responses were observed at other DIRT sites, with SOC stocks varying between 0 and 60% of the pre-disturbance steady state after 5+ years of treatment (Fig. 6a; Supplementary Figs. 10a and 11).

The microbial models without density-dependence are unable to capture the range of SOC responses observed in the DIRT experiments (Figs. 5a and 6a), as a direct consequence of their model structure. Similarly, the linear model structure predicts that the SOC steady state will always change proportionally to the change in C inputs. These two model formulations are thus limited in the SOC responses that they can predict, and do not match observations given an increase in total C inputs. In contrast, the microbial models with density-dependence can, depending on the value of *β*, capture a change in the long-term SOC stocks that is within the range observed from experiments (dark blue line, Figs. 5a and 6a). Long-term (> 5 years) MBC measurements and replicates were not available from most DIRT plots and, therefore, the comparison with MBC model output for a doubling of C inputs was inconclusive (Supplementary Table 2; Supplementary Fig. 12a).

### Removing C inputs in models and experiments

We compared SOC and MBC projections for a complete removal of C inputs (Fig. 5b, d) to the analogous treatments in the DIRT and LTBF experiments (Fig. 6b; Supplementary Fig. 10). We synthesized results from 18 different sites with measurements spanning 5–80 years (Supplementary Tables 2 and 3)^30, 31, 45–47^ and summarized the observed SOC and MBC responses (Fig. 6b; Supplementary Figs. 11 and 12b). The microbial models without density-dependence (*β* = 1) predicted a rapid decrease in MBC, where MBC completely died out within 10 years, despite remaining SOC stocks; only ~ 20% of SOC stocks were lost by the time MBC declined to zero (Fig. 5b; Supplementary Fig. 13). In contrast, the linear model showed a gradual decrease in all pools, approaching zero after 80+ years. While the microbial models with density-dependent turnover (*β* = 2) gave similar predictions for C-input removal to the linear model, they experienced a faster SOC decline in the first two decades that became more gradual over time (Fig. 5b). All of the long-term C-input removal sites showed a gradual decrease in SOC stocks (Fig. 6b), where most sites reached a period of slower decomposition by 50 years, and MBC decreased but did not die out (Supplementary Fig. 12b; Supplementary Tables 2 and 3).

## Discussion

Due to the important contribution of SOC to the terrestrial C budget following changes in plant productivity, it is crucial that soil C models are able to capture the transient and steady-state dynamics of long-term C-input manipulations in the field. Our results show that across the range of model complexities investigated, microbial models without density-dependent microbial turnover (i.e., *β* = 1) return to their pre-disturbance SOC steady state following a sustained increase in total C inputs, for any increase in inputs (Fig. 4; Supplementary Fig. 7). This behavior is a direct consequence of the microbial model structure, which also results in damped decadal oscillations for most parameter sets reported in the literature (Fig. 2). The decoupling of C inputs and long-term SOC stocks has been observed in several modeling studies that effectively used *β* = 1^20, 23, 25^. The proportionality between C inputs and long-term MBC (and the resulting insensitivity of long-term SOC) implies that MBC is only limited by the C-input rate (Supplementary Fig. 7), but in fact it can also be limited by community-level regulatory mechanisms^38–41^. In microbial models with *β* = 1, a change in C inputs drives a proportional change in the long-term ratio of MBC to SOC (*C*_B_/*C*_S_; Supplementary Note 1), in contrast to global observations that suggest this ratio is confined to a rather narrow range^48^.

Increasing the density-dependence *β* > 1 strengthens community-level regulation of MBC, and therefore limits how large (and how quickly) the MBC pool can grow or decline (Fig. 3). The case of *β* = 2 has been widely used in modeling population dynamics, including past biogeochemical models^18^, and it is a logical modification: microbes experience proportionally more mortality (e.g., due to competition, space constraints, disease, and production of toxins) as their concentrations increase. We show that a value of 1 < *β* < 2 imparts a moderate SOC sensitivity to C inputs and acts to reduce oscillations (Figs. 2 and 3). We expect the effective value of *β* to vary between biomes, and potentially with depth, depending on the strength of microbial community-level regulation that arises from biotic and abiotic limitations. The high sensitivity of microbial model predictions to changes in *β* highlights the need for experiments that quantify its value and its relation to explicit underlying regulatory mechanisms in different soils. Meta-analyses exploring MBC/SOC ratios^48, 49^ and emergent scaling relationships of microbial function^50–53^ corroborate the density-dependent turnover (*β* > 1) formulation and are invaluable for constraining the value of *β* in future modeling studies.

Plant-input manipulation experiments show that long-term measured SOC stocks are, in fact, sensitive to C-input rate in the field, contrary to the predicted SOC stocks from most microbial models (Figs. 5a and 6a). Following an increase in C inputs, microbial models without density-dependent turnover (i.e., *β* = 1) overestimate the response of MBC and, thus, underestimate the response of SOC (Figs. 5a, c and 6a). This suggests that a mechanism limiting the size of the microbial pool is necessary, and consistent with a density-dependent turnover formulation. Furthermore, in microbial models with *β* = 1, MBC disappears within 5–10 years of ceasing plant inputs (Fig. 5d), and SOC decreases too quickly to a nonzero value and is invariant once microbes die out (Figs. 5b and 6b). The complete loss of MBC after cessation of C inputs was also predicted by a microbial model (with *β* = 1) that included organo-mineral interactions and microbial physiology^54^ (similar to the five-pool model; Fig. 1d), but this prediction had not been compared to data. In contrast, the empirical evidence synthesized here^55–57^ suggests that SOC and MBC continue to decrease slowly over decades (Fig. 6b; Supplementary Fig. 12). Density-dependent turnover in microbial models slows the decay of SOC and MBC such that they persist for 80+ years, better matching observations and further corroborating the need for a density-dependent turnover formulation.

We note that other microbial model formulations exist in the literature that behave differently than the class of models presented in this study. For example, the so-called “reverse Michaelis–Menten” microbial model^14, 19, 58^—a class of equilibrium chemistry approximation kinetics^59, 60^—predicts a slight sensitivity of the SOC steady state to C inputs, but the MBC steady state is again directly proportional to the C-input rate^26^. While this formulation dampens oscillations^26^, the decadal oscillations do persist, and the long-term response to C-input manipulations has not been evaluated. Another example is the microbial-mineral carbon stabilization (MIMICS) model^22, 61^. Recently, the microbial turnover rate in MIMICS was modified by a factor of $\sqrt I$ (where *I* denotes C inputs) to better explain litter decomposition observations^62^. Interestingly, explicit density-dependent microbial turnover in microbial models can be shown to impart this same modification on the microbial turnover rate over long timescales; for *β* = 2, ${C_{\rm{B}}}\sim \sqrt I$ and thus microbial turnover is proportional to $\sqrt I \cdot {C_{\rm{B}}}$.

Dormancy is another mechanism that has recently been incorporated in microbial models and has shown promise in better matching respiration seasonality and moisture sensitivity^63^. A dormancy formulation allows microbes to switch from an active state (in which they decompose SOC and proliferate) to a dormant state^63, 64^. In the case of substrate limitation or starvation following C-input removal, this mechanism may help preserve MBC, albeit in a dormant state, over longer periods of time than in microbial models without dormancy. This MBC would then be available to respond rapidly to future increases in C inputs and favorable environmental conditions, as observed in field and laboratory experiments^46, 56, 57^. However, if dormancy approaches zero when C inputs to the soil are increased^63, 64^, the resulting model approaches the microbial models without dormancy and will still be insensitive to increases in C inputs. Thus, even with dormancy, future formulations are likely to require a density-dependent turnover, either in the microbial mortality term or in the transition between active and dormant microbial states.

When connecting global change experiments to model predictions, variable changes in plant C inputs over time (as opposed to step changes in many manipulations) may prevent SOC from reaching a steady state on relevant time scales. Thus, models must accurately capture transient dynamics, where community-level regulation (i.e., here density-dependence with *β* = 2) plays a key role. Step-change scenarios are also highly relevant for studying the impacts of land-use change. While we have focused on C-only manipulations in this study, more complex models that explicitly represent nutrients may be necessary to capture other biogeochemical interactions; e.g., addition/limitation of nitrogen (N) and phosphorus (P)^43^. However, our results on SOC model stability and sensitivity to C inputs are robust and relevant to more complex C–N or C–N–P models as well, since a similar representation of key processes is used in such models.

Given the significant and growing interest in developing microbial models for predicting soil C dynamics over large spatiotemporal scales, it is imperative to compare proposed models and validate model behaviors against observations. This validation is particularly important in light of the significant divergence among soil C model predictions to future temperature and C-input change, which have far-reaching global implications^65, 66^. Here we diagnosed structural and parametric differences between models ranging in complexity, and synthesized a novel data set from long-term litter manipulation experiments to evaluate the models and validate our hypothesis. Analyzing the underlying mechanisms of simple microbial models allowed us to identify the source of, and potential solution to, behavior deemed unrealistic in recent studies. Namely, a model formulation with density-dependent microbial turnover (*β* > 1; arising from competition, space, or other community-level regulation mechanisms) reduced oscillations and resulted in a realistic sensitivity of long-term SOC stocks to (and a decoupling of the MBC/SOC ratio from) C inputs. All else equal, the proposed formulation implies larger SOC storage associated with expected CO_2_-fertilization-driven increases in C inputs over the coming century compared to microbial models with *β* = 1. Moving forward, we encourage the validation of subcomponents of increasingly complex microbial models before they are applied at field or larger scales, and a concerted effort to identify additional metrics and datasets to benchmark mechanistic models.

## Methods

### SOC model formulations

In this study we compare four archetypal SOC models that range in complexity (Fig. 1). Such models are common in recent literature^14, 20, 54, 61^, and require careful theoretical investigation of model behavior prior to implementation on larger spatial scales. To understand their behavior in response to C-input perturbations, we have dissected model components and added complexity incrementally, beginning from the simplest two-pool microbial model. This two-pool model has appeared in many studies (e.g., in refs ^21, 25, 35^), and here we adopt parameters from the version in ref ^35^. The C pools represented in the two-pool model (Fig. 1a) are SOC (denoted *C*_S_ in subsequent equations) and MBC (denoted *C*_B_). The two-pool microbial model can be represented as follows,

3 $$\frac{{{\rm{d}}{C_{\rm{S}}}}}{{{\rm{d}}t}} = I - \left( {\frac{{{V_{{\rm{max,U}}}}\cdot{C_{\rm{B}}}\cdot{C_{\rm{S}}}}}{{{K_{{\rm{M,U}}}} + {C_{\rm{S}}}}}} \right) + {k_{\rm{B}}}\cdot{C_{\rm{B}}}^\beta $$

4 $$\frac{{{\rm{d}}{C_{\rm{B}}}}}{{{\rm{d}}t}} = \varepsilon \cdot\left( {\frac{{{V_{{\rm{max,U}}}}\cdot{C_{\rm{B}}}\cdot{C_{\rm{S}}}}}{{{K_{{\rm{M,U}}}} + {C_{\rm{S}}}}}} \right) - {k_{\rm{B}}}\cdot{C_{\rm{B}}}^\beta $$

where *I* is the carbon input rate, *V*_max,U_ the maximum microbial assimilation rate of SOC, *K*_M,U_ the half-saturation for assimilation, *k*_B_ the microbial mortality (turnover) rate constant, and *ε* the microbial C-use efficiency (see Supplementary Table 1 for units and details). We also note that *β* (the density-dependence exponent) is proposed as a modification in this study, but has been equal to 1 (i.e., microbial mortality is directly proportional to *C*_B_) in previous studies (e.g., refs ^21, 25, 35^). The condition ${k_{\rm{B}}} \le C_{\rm{B}}^{1 - \beta }$ must hold for any value of *β*, such that the mass balance constraint on microbial turnover, ${k_{\rm{B}}}\cdot C_{\rm{B}}^\beta \le {C_{\rm{B}}}$, is satisfied; for *β* = 2, this implies that ${k_{\rm B}} \le \left( \frac{ 1 }{ {{C_{\rm{B}}}} }\right)$.

We compare the dynamics of this two-pool microbe model to a four-pool microbe-enzyme model in which we have added carbon pools for dissolved organic carbon (DOC; denoted *C*_D_ in all subsequent equations) and enzymatic carbon (denoted *C*_E_). This structure allows for the separation of enzymatic decomposition and microbial uptake of organic carbon (Fig. 1c). The four-pool microbial model can then be written as

5 $$\frac{{{\rm{d}}{C_{\rm{S}}}}}{{{\rm{d}}t}} = f\cdot I - \left( {\frac{{{V_{{\rm{max}}}}\cdot{C_{\rm{E}}}\cdot{C_{\rm{S}}}}}{{{K_{\rm{M}}} + {C_{\rm{S}}}}}} \right) + {a_{{\rm{BS}}}}\cdot{k_{\rm{B}}}\cdot{C_{\rm{B}}}^\beta $$

6 $$\frac{{{\rm{d}}{C_{\rm{D}}}}}{{{\rm{d}}t}} = \left( {1 - f} \right)\cdot I + \left( {\frac{{{V_{{\rm{max}}}}\cdot{C_{\rm{E}}}\cdot{C_{\rm{S}}}}}{{{K_{\rm{M}}} + {C_{\rm{S}}}}}} \right) - \left( {\frac{{{V_{{\rm{max,U}}}}\cdot{C_{\rm{B}}}\cdot{C_{\rm{D}}}}}{{{K_{{\rm{M,U}}}} + {C_{\rm{D}}}}}} \right) + \left( {1 - {a_{{\rm{BS}}}}} \right)\cdot{k_{\rm{B}}}\cdot{C_{\rm{B}}}^\beta + {r_{\rm{E}}}\cdot{C_{\rm{E}}}$$

7 $$\frac{{{\rm{d}}{C_{\rm{B}}}}}{{{\rm{d}}t}} = \varepsilon \cdot \left( {\frac{{{V_{{\rm{max,U}}}}\cdot{C_{\rm{B}}}\cdot{C_{\rm{D}}}}}{{{K_{{\rm{M,U}}}} + {C_{\rm{D}}}}}} \right) - {k_{\rm{B}}}\cdot{C_{\rm{B}}}^\beta - {r_{\rm{p}}}\cdot{C_{\rm{B}}}$$

8 $$\frac{{{\rm{d}}{C_{\rm{E}}}}}{{{\rm{d}}t}} = {r_{\rm{p}}}\cdot{C_{\rm{B}}} - {r_{\rm{E}}}\cdot{C_{\rm{E}}}$$

where *V*_max_ is the maximum enzymatic decomposition rate of SOC, *K*_M_ the half-saturation for decomposition, *f* the fraction of inputs that enters the SOC pool, *a*_BS_ the fraction of microbial turnover into SOC, *r*_E_ the enzyme turnover rate constant, and *r*_p_ the enzyme production rate constant.

We next include mineral sorption of DOC in a five-pool microbial model, where DOC can reversibly bind to mineral surfaces forming a mineral-associated C pool (denoted *C*_q_) that is protected from microbial uptake (Fig. 1d). This can be represented as

9 $$\frac{{{\rm{d}}{C_{\rm{S}}}}}{{{\rm{d}}t}} = f\cdot I - \left( {\frac{{{V_{{\rm{max}}}}\cdot{C_{\rm{E}}}\cdot{C_{\rm{S}}}}}{{{K_{\rm{M}}} + {C_{\rm{S}}}}}} \right) + {a_{{\rm{BS}}}}\cdot{k_{\rm{B}}}\cdot{C_{\rm{B}}}^\beta $$

10 $${\frac{{{\rm{d}}{C_{\rm{D}}}}}{{{\rm{d}}t}}} = 	\, {\left( {1 - f} \right)\cdot I + \left( {\frac{{{V_{{\rm{max}}}}\cdot{C_{\rm{E}}}\cdot{C_{\rm{S}}}}}{{{K_{\rm{M}}} + {C_{\rm{S}}}}}} \right) - \left( {\frac{{{V_{{\rm{max,U}}}}\cdot{C_{\rm{B}}}\cdot{C_{\rm{D}}}}}{{{K_{{\rm{M,U}}}} + {C_{\rm{D}}}}}} \right) + \left( {1 - {a_{{\rm{BS}}}}} \right)\cdot{k_{\rm{B}}}\cdot{C_{\rm{B}}}^\beta } \\ 	 \, {{ + {r_{\rm{E}}}\cdot{C_{\rm{E}}} - {k_{{\rm{ads}}}}\cdot{C_{\rm{D}}}\left( {{Q_{{\rm{max}}}} - {C_q}} \right) + {k_{{\rm{des}}}}\cdot{C_q}} }$$

11 $$\frac{{{\rm{d}}{C_{\rm{B}}}}}{{{\rm{d}}t}} = \varepsilon \cdot\left( {\frac{{{V_{{\rm{max,U}}}}\cdot{C_{\rm{B}}}\cdot{C_{\rm{D}}}}}{{{K_{{\rm{M,U}}}} + {C_{\rm{D}}}}}} \right) - {k_{\rm{B}}}\cdot{C_{\rm{B}}}^\beta - {r_{\rm{p}}}\cdot{C_{\rm{B}}}$$

12 $$\frac{{{\rm{d}}{C_{\rm{E}}}}}{{{\rm{d}}t}} = {r_{\rm{p}}}\cdot{C_{\rm{B}}} - {r_{\rm{E}}}\cdot{C_{\rm{E}}}$$

13 $$\frac{{{\rm{d}}{C_q}}}{{{\rm{d}}t}} = {k_{{\rm{ads}}}}\cdot{C_{\rm{D}}}\cdot({Q_{{\rm{max}}}} - {C_q}) - {k_{{\rm{des}}}}\cdot{C_q}$$

where *k*_ads_ is the adsorption rate constant, *k*_des_ the desorption rate constant, and *Q*_max_ the maximum DOC adsorption capacity, i.e., the mineral surface area available for organo-mineral interactions.

Finally, we compare these microbial models to a three-pool linear, first-order model (Fig. 1b). The C pools represented include SOC, DOC, and MBC, and the transfer of C between these pools is directly proportional only to the pool where it originates^35, 67^. The three-pool linear model can be written as follows,

14 $$\frac{{{\rm{d}}{C_{\rm{S}}}}}{{{\rm{d}}t}} = f\cdot I + {f_{\rm{D}}}\cdot{k_{\rm{D}}}\cdot{C_{\rm{D}}} + {f_{\rm{B}}}\cdot{f_{{\rm{B}} \to {\rm{S}}}}\cdot{k_{\rm{B}}}\cdot{C_{\rm{B}}} - {k_{\rm{S}}}{C_{\rm{S}}}$$

15 $$\frac{{{\rm{d}}{C_{\rm{D}}}}}{{{\rm{d}}t}} = \left( {1 - f} \right)\cdot I + {f_{\rm{S}}}\cdot{k_{\rm{S}}}\cdot{C_{\rm{S}}} + {f_{\rm{B}}}\cdot(1 - {f_{{\rm{B}} \to {\rm{S}}}}){k_{\rm{B}}}\cdot{C_{\rm{B}}} - {k_{{\rm{uptake}}}}\cdot{C_{\rm{D}}} - {k_{\rm{D}}}\cdot{C_{\rm{D}}}$$

16 $$\frac{{{\rm{d}}{C_{\rm{B}}}}}{{{\rm{d}}t}} = {k_{{\rm{uptake}}}}\cdot{C_{\rm{D}}} - {k_{\rm{B}}}\cdot{C_{\rm{B}}}$$

where *k*_S_ is the SOC decay rate constant, *k*_D_ the DOC decay rate constant of DOC, *k*_B_ the MBC turnover rate constant, *f*_S_ the fraction of SOC entering the DOC pool, *f*_D_ the fraction of DOC entering the SOC pool, *f*_B_ the fraction of MBC turnover reentering the C pools (similar to a C-use efficiency), *f*_B→S_ the fraction of retained MBC turnover C that enters the SOC pool, and *k*_uptake_ the DOC uptake rate constant.

### Density-dependent microbial turnover

In population ecology, density-dependent processes, motivated by community-level interactions, are regulated by the size of the population itself. For example, this may take the form of density-dependent microbial turnover that effectively limits the potential size of a population. A typical formulation is logistic growth

17 $$\frac{{{\rm{d}}{C_{\rm{B}}}}}{{{\rm{d}}t}} = r \cdot {C_{\rm{B}}} \cdot \left( {1 - \frac{{{C_{\rm{B}}}}}{K}} \right)$$

where *r* is the growth rate and *K* is the carrying capacity of the population. In this equation, at low populations, the unimpeded growth is modeled as the first term ($r \cdot {C_{\rm{B}}}$), which results in exponential growth. As the population grows, however, the second term ($- r \cdot \frac{{C_{\rm{B}}^2}}{K}$) begins to overtake the first term, thereby limiting the population size. This is an intuitive regulation of the population size to some carrying capacity, resulting from competition, space constraints, and other density-dependent controls such as disease and toxicity.

Equation (17) can be rearranged to have the same form as the microbial models presented herein and used in the literature. For example, from Eq. (4) with *β* = 2, we have

18 $$\frac{{{\rm{d}}{C_{\rm{B}}}}}{{{\rm{d}}t}} = \varepsilon \cdot \left( {\frac{{{V_{{\rm{max,U}}}} \cdot {C_{\rm{B}}} \cdot {C_{\rm{S}}}}}{{{K_{{\rm{M,U}}}} + {C_{\rm{S}}}}}} \right) - {k_{\rm{B}}} \cdot {C_{\rm{B}}}^2$$

where the logistic growth parameters can be represented as

19 $$r = \varepsilon \cdot \left( {\frac{{{V_{{\rm{max,U}}}} \cdot {C_{\rm{S}}}}}{{{K_{{\rm{M,U}}}} + {C_{\rm{S}}}}}} \right)$$

and $\frac{r}{K} = {k_{\rm{B}}}$ so that

20 $$K = \frac{\varepsilon }{{{k_{\rm{B}}}}} \cdot \left( {\frac{{{V_{{\rm{max,U}}}} \cdot {C_{\rm{S}}}}}{{{K_{{\rm{M,U}}}} + {C_{\rm{S}}}}}} \right).$$

Here the growth rate and carrying capacity depend on the substrate availability (*C*_S_) and thus, unlike most existing microbial models, microbes experience an additional constraint on population size.

We note that the parameter *β* = 2 gives logistic growth, but this parameter need not be equal to 2. In fact, any value > 1 will impart a density-dependent microbial turnover rate (i.e., more mortality when populations are large) that will limit population sizes.

### Parameter values

For the two-, three-, and four-pool models, we adapted parameter values from ref. ^35^, where the three models were fit to obtain comparable steady-state solutions among corresponding C pools in each model. We used empirical rate constants for the organo-mineral interactions in the five-pool model^68, 69^ and matched the steady-state solutions of the other models. We note that a subset of the parameters from ref. ^35^ was derived from lab incubations in the literature and the remaining parameters were fit to attain reasonable field C pool steady-state solutions. However, rates measured in the lab may not be representative of process rates in the field or at ecosystem scales. Although we use this particular set of parameters in our numerical simulations, our analytical work shows that our conclusions about model behavior are robust to the choice of parameter values. Additional details about the parameter values are summarized in Supplementary Table 1.

### Analytical and numerical evaluation

We analytically derived the steady-state solutions of each of the four models, with and without density-dependent microbial turnover, by setting all $\frac{{{\rm{d}}{C_i}\left( t \right)}}{{{\rm{d}}t}} = 0$ (where *C*_*i*_(*t*) is the concentration of the *i*th component in time) and solving the corresponding algebraic equations. We then analyzed the dependence (or lack thereof) of the model steady-state solutions on the rate of C inputs. Furthermore, we simulated all four models to analyze their diverse transient responses to increased and decreased C inputs.

We also performed a stability analysis for each of the four models to understand their behavior and explain the emergence of oscillations with select model structures and parameter sets. We linearized each model using a multivariable Taylor expansion, forming the Jacobian (*J*) matrix from the first-order partial derivatives evaluated at equilibrium. The eigenvalues (*λ*_*j*_) of the matrix *J* characterize the stability of the system, since the solution can be written as a superposition of exponential ${{\rm{e}}^{{\lambda _j}t}}$ terms, where *t* denotes time. The eigenvalues were calculated for each model to understand the dynamics near equilibrium, where for *λ*_*j*_ = *α*_*j*_ ± *γ*_*j*_ *i* the following scenarios can be summarized: (1) *γ*_*j*_ = 0, *α*_*j*_ < 0 corresponds to a stable mode and *α*_*j*_ > 0 to an unstable mode; and (2) *γ*_*j*_ ≠ 0, *α*_*j*_ < 0 corresponds to a damped (diminishing) oscillation, *α*_*j*_ > 0 to an unstable (increasing) oscillation, and *α*_*j*_ = 0 to a persistent oscillation with a period of $\frac{{2\pi }}{{{\gamma _j}}}$. The damping ratio (*ζ*_*j*_) can then be defined as

21 $${\zeta _j} = \frac{{ - {\alpha _j}}}{{\sqrt {{\alpha _j}^2 + {\gamma _j}^2} }}$$

for each eigenvalue *λ*_*j*_, where *ζ* = 1 signifies a stable mode, 0 < *ζ* < 1 signifies damped oscillations, and *ζ* < 0 signifies an unstable equilibrium^29, 70^. In the case of the two-pool microbial model, there is a single complex conjugate pair of eigenvalues and the corresponding value of *ζ* was calculated as a function of the model parameters (Fig. 2). We assign the damping ratio of the dominant mode (smallest *ζ*) as the damping ratio of the linearized system and use this as a metric to illustrate the stability of the models under different parameter sets and with/without density-dependent microbial turnover.

### Long-term C-input simulations and observations

We simulated the response of the models to a doubling (2×; 100% increase) and removal (0×; 100% decrease) of C inputs, following the manipulations of the long-term DIRT and LTBF experiments. We then compared the model results to our synthesis of long-term observations from DIRT and LTBF studies. We compiled DIRT and LTBF data of SOC and MBC from 18 sites with 24 total (6 doubled litter and 18 litter removal) manipulations, each across several time points ranging from 5 to 80 years (summarized in Supplementary Tables 2 and 3). We provide a summary of the findings across these sites, but focus our model-data comparison on experiments with over 20 years of measurements; for example, the 50+ year Noe and Wingra Woods Wisconsin DIRT experiment^30^ and the 50+ year LTBF experiments^31^. We also synthesized MBC observations^55, 56^, which are summarized in Supplementary Fig. 12.

### Data availability

Source code and data are available from the corresponding authors on request. Details about the data and the data sources are reported in Supplementary Tables 2 and 3.

## Electronic supplementary material

Supplementary Information
