# Publisher Correction: Microbial community-level regulation explains soil carbon responses to long-term litter manipulations

**DOI:** 10.1038/s41467-017-02134-7

**Published:** 2018-01-09

**Authors:** Katerina Georgiou, Rose Z. Abramoff, John Harte, William J. Riley, Margaret S. Torn

**Affiliations:** 1 Department of Chemical and Biomolecular Engineering, University of California, Berkeley, CA 94720 USA; 2 Climate and Ecosystem Sciences Division, Lawrence Berkeley National Laboratory, Berkeley, CA 94720 USA; 3 Energy and Resources Group, University of California, Berkeley, CA 94720 USA

*Nature Communications* **8**:1223 10.1038/s41467-017-01116-z; published online: 31 October 2017

The original PDF version of this Article contained an error in Table 1. On the right-hand side of the third row, the third equation was missing a *β* as an exponent on the first *C*_B_, and incorrectly read:

$$k_{\rm B}{{C}}_{{\rm B}} + r_{\rm p}{{C}}_{{\rm B}} = \frac{{\varepsilon \cdot {{\rm I}}}}{{\left( {1 - \varepsilon } \right)}}$$

The correct form of the equation is as follows:

$$k_{\rm B}{{C}}_{{\rm B}} \, ^{{\beta }} + r_{\rm p}{{C}}_{{\rm B}} = \frac{{\varepsilon \cdot {{\rm I}}}}{{\left( {1 - \varepsilon } \right)}}$$

This has now been corrected in the PDF version of the Article. The HTML version was correct from the time of publication.

