# Supplementary material for: Microbial community-level regulation explains soil carbon responses to long-term litter manipulations
Source: Nat Commun. 2017 Oct 31;8:1223. doi: 10.1038/s41467-017-01116-z (PMC5663850; doi:10.1038/s41467-017-01116-z)
Supplement: Supplementary file 1 — Supplementary Information [file 41467_2017_1116_MOESM1_ESM.pdf]

Supplementary Note 1.

In microbial models without density-dependent microbial turnover ($\beta = 1$), the steady-state ratio of microbial biomass carbon (MBC; C_B) to soil organic carbon (SOC; C_S) is proportional to the C input rate, as a result of the respective proportionality and insensitivity of long-term MBC and SOC to total C inputs. For the 2-pool microbial model with $\beta = 1$, the steady-state ratio of MBC/SOC can be written as follows

$$\frac{C_B}{C_S} = \frac{\left(\frac{\varepsilon \cdot I}{(1-\varepsilon) \cdot k_B}\right)}{\left(\frac{K_{M,U} \cdot k_B}{\varepsilon \cdot V_{max,U} - k_B}\right)}, \tag{22}$$

which is clearly proportional to the total C input rate; that is, $\frac{C_B}{C_S} \propto I^1$. In contrast, for microbial models with density-dependent microbial turnover ($\beta > 1$), the proportionality between the steady-state MBC/SOC ratio and C input rate decreases. Consider the 2-pool microbial model with $\beta = 2$, for example. In this case, the steady-state ratio of MBC/SOC can be written as

$$\frac{C_B}{C_S} = \frac{\varepsilon \cdot V_{max,U} - \left(\frac{\varepsilon \cdot I \cdot k_B}{(1-\varepsilon)}\right)^{1/2}}{K_{M,U} \cdot k_B}. \tag{23}$$

This quantity has two limiting cases of sensitivity to the total C input rate, depending on the relative magnitude of the two terms in the numerator. When $(\varepsilon \cdot V_{max,U}) > \left(\frac{\varepsilon \cdot I \cdot k_B}{(1-\varepsilon)}\right)^{1/2}$, which is the case for parameter sets used in the literature and given in Supplementary Table 1, then the steady-state MBC/SOC ratio is independent of the C input rate; that is, $\frac{C_B}{C_S} \propto I^0$. This decoupling of the steady-state MBC/SOC ratio from the C input rate in microbial models with density-dependence ($\beta > 1$) is corroborated by global observations showing that this ratio is confined to a narrow range around 1-2% [1,2]. First-order models, such as the 3-pool linear model in Fig. 1b, also predict a steady-state MBC/SOC ratio that is independent of the total C input rate; however, this is simply because each pool changes proportionally to the total C inputs. This proportionality of SOC stocks to the change in total C inputs was not observed in the DIRT experiments (Fig. 6). Comparing the predicted long-term MBC/SOC ratio, in addition to individual pool sizes, to observations can be a useful metric for validating models and constraining the value of β in future studies.

Supplementary Note 2.

In the 4-pool microbial model, the SOC steady state is insensitive to changes in C inputs when both SOC inputs (I_S) and DOC inputs (I_D) – i.e., the total I in equations (5-8) – are changed by the same proportion (Supplementary Figs. 6 and 7). This perturbation in total I causes an oscillatory response in all C pools (shown for SOC and MBC) with a period of ~20 years. The case where only I_S is doubled also results in oscillations; however, an increase in the SOC steady state of $+2.9\%$ is observed (Supplementary Fig. 6). Conversely, when only I_D is doubled, we observe that oscillations are largely dampened and a decrease in the SOC steady state of -5.5% is observed (Supplementary Fig. 6). These responses were markedly different than those of the 4-pool microbial model with density-dependent microbial turnover ($\beta = 2$) and the 3-pool linear model for a doubling of I_S, I_D, or both (Supplementary Fig. 6).

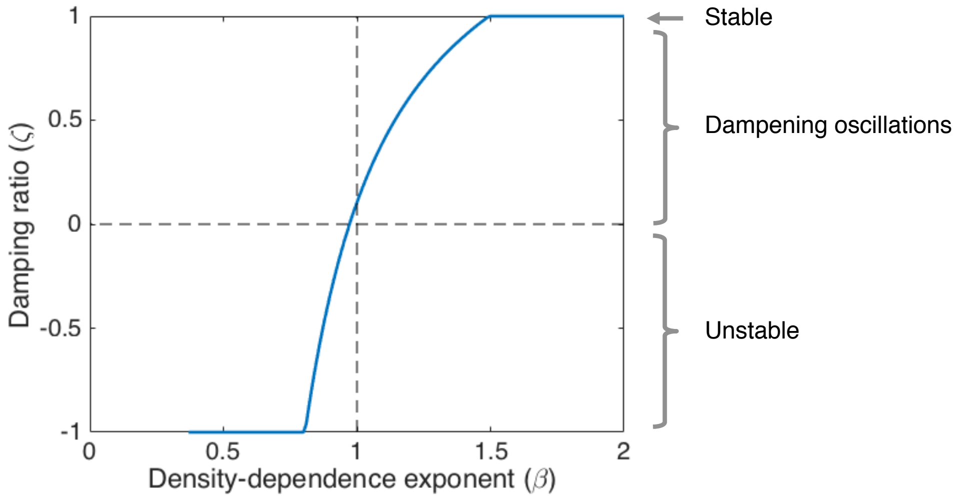

Supplementary Figure 1: **Stability of the 2-pool microbial model for a range of density-dependent microbial turnover exponents.** The damping ratio (ζ; defined in equation (21)) illustrates the degree of oscillatory behavior that the system will display following a perturbation. The system is increasingly stable with a larger density-dependent microbial turnover exponent (β), where for $\beta \geq 1.5$, a stable node ($\zeta = 1$) is achieved.

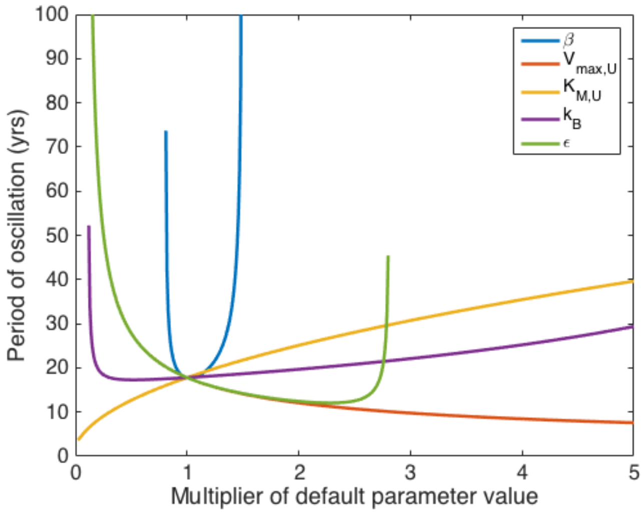

Supplementary Figure 2: **Period of oscillation of the 2-pool microbial model as a function of the model parameters.** This depicts the degree of oscillatory behavior of the linearized system near its steady-state following a perturbation. The period of oscillation ($2\pi/\gamma$, where $\gamma = Im(\lambda)$ for eigenvalue λ) depends on the model parameters. Parameters are defined in Supplementary Table 1.

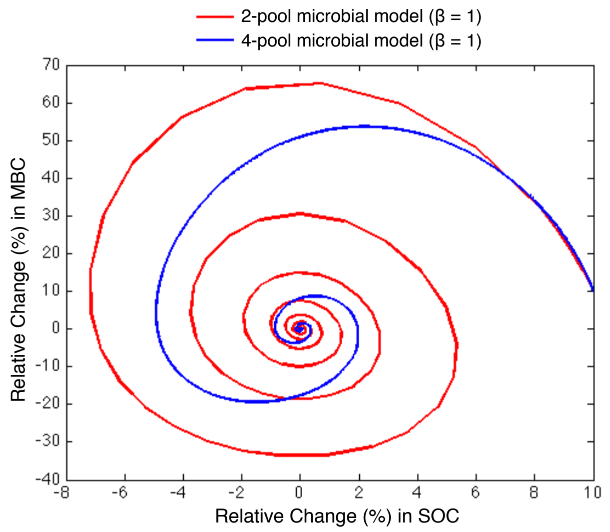

<u>Supplementary Figure 3:</u> Stability and periodicity of the 2- and 4-pool microbial models without density-dependent microbial turnover. Phase portrait of the relative change of soil organic carbon (SOC) and microbial biomass carbon (MBC) in response to a 10% change in initial conditions. For parameter sets commonly used in the literature (Supplementary Table 1), the 2-pool microbial model has a pair of complex eigenvalues ($\lambda_j = \alpha_j \pm \gamma_j i$) with $\alpha_j < 0, \gamma_j \neq 0$ and the 4-pool microbial model has a complex pair of λ and 2 negative real λ with $\alpha_j < 0, \gamma_j \neq 0$. Thus, both models exhibit damped oscillations. Similarly the 5-pool microbial model has a complex pair of λ and 3 negative real λ with $\alpha_j < 0, \gamma_j \neq 0$, again exhibiting damped oscillations. In contrast, the 3-pool linear model has all 3 negative, real λ with $\alpha_j < 0, \gamma_j = 0$, which signifies a stable node. The 2-pool microbial model is the most oscillatory, as expected, since its damping ratio is closest to zero.

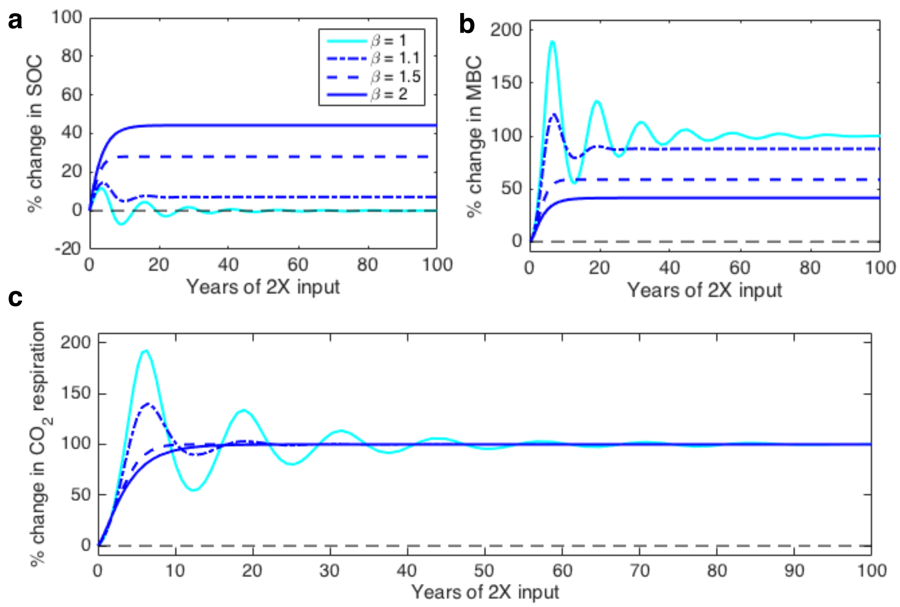

<u>Supplementary Figure 4:</u> Response of the 2-pool microbial model to a step 2X of inputs for a range of β using standard parameter values. Percent change of modeled **(a)** SOC, **(b)** MBC, and **(c)** CO_2, where $\beta > 1$ corresponds to a microbial model with density-dependent microbial turnover. The standard value of carbon use efficiency (CUE; ε) at 20°C is 0.31, as in Supplementary Table 1.

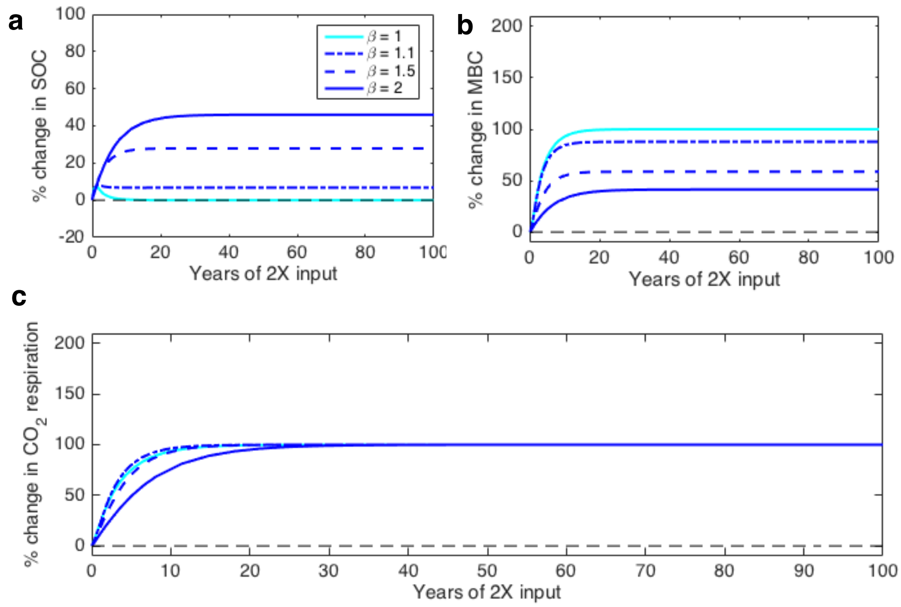

<u>Supplementary Figure 5:</u> Response of the 2-pool microbial model to a step 2X of inputs for a range of β using a greater carbon use efficiency than the standard value. Percent change of modeled **(a)** SOC, **(b)** MBC, and **(c)** CO_2, where $\beta > 1$ corresponds to a microbial model with density-dependent microbial turnover. Here a carbon use efficiency (CUE; ε) at 20°C of 0.90 was used. Although this CUE may be unrealistically high under most soil conditions, this illustrates how the transient dynamics are dependent on the parameter values, while the steady-state behavior is largely a consequence of the model structure. Here oscillations are diminished with larger CUE (as compared to Supplementary Fig. 4). Varying the parameter K_m (not shown) results in a response curve that is less steep at early times, especially for larger β exponents.

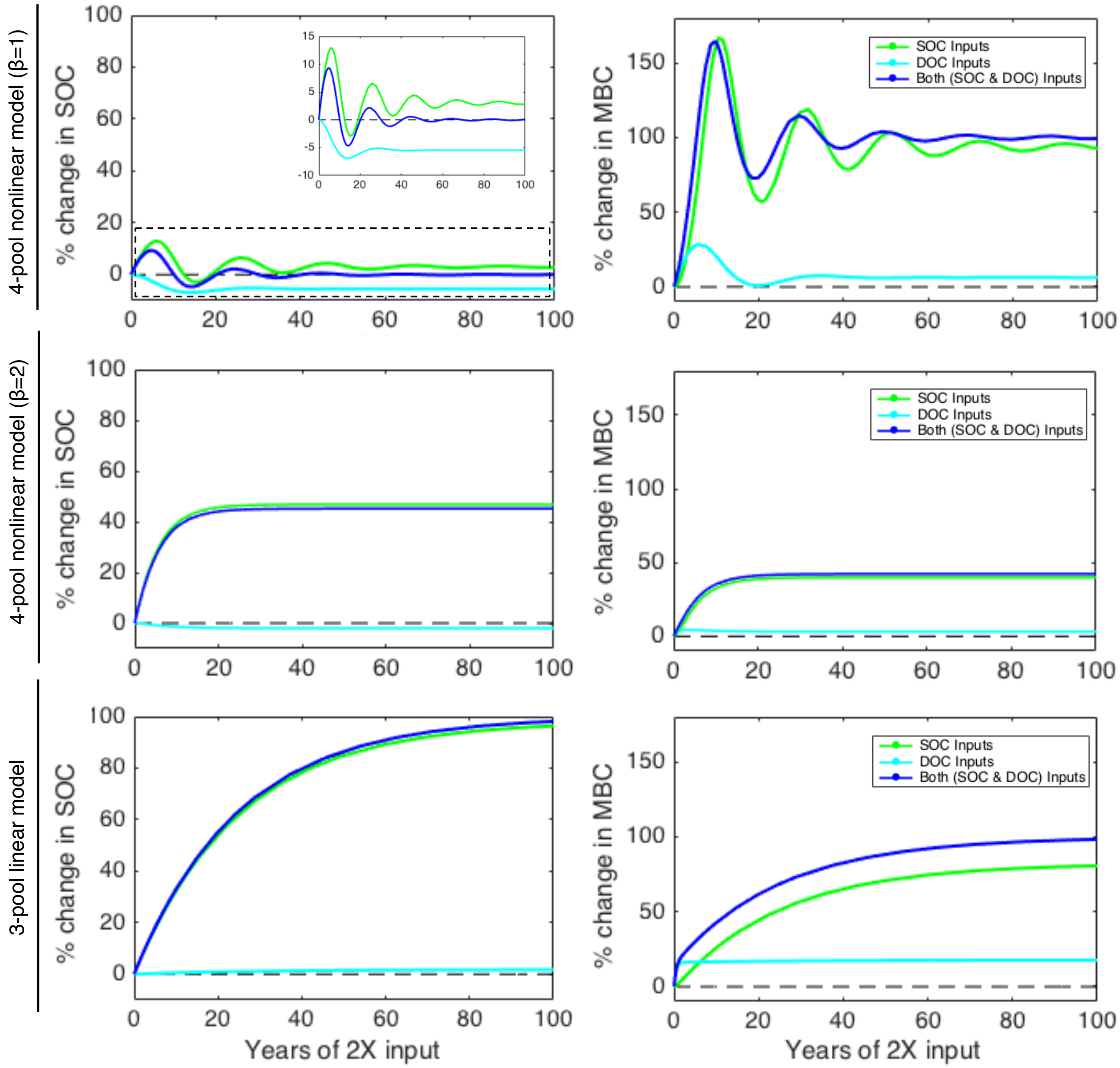

<u>Supplementary Figure 6:</u> Response of the 4-pool microbial model with and without density-dependence and the linear 3-pool model to a doubling of inputs. SOC, DOC, or both inputs are individually doubled (2X) in each model. *Left panels:* Percent change in SOC with time. For the 4-pool microbial model, the SOC steady-state increases with 2X SOC, decreases with 2X DOC, and is insensitive to 2X SOC + 2X DOC. The inset plot zooms into the dashed box. *Right panels:* Percent change in microbial biomass carbon (MBC) with time.

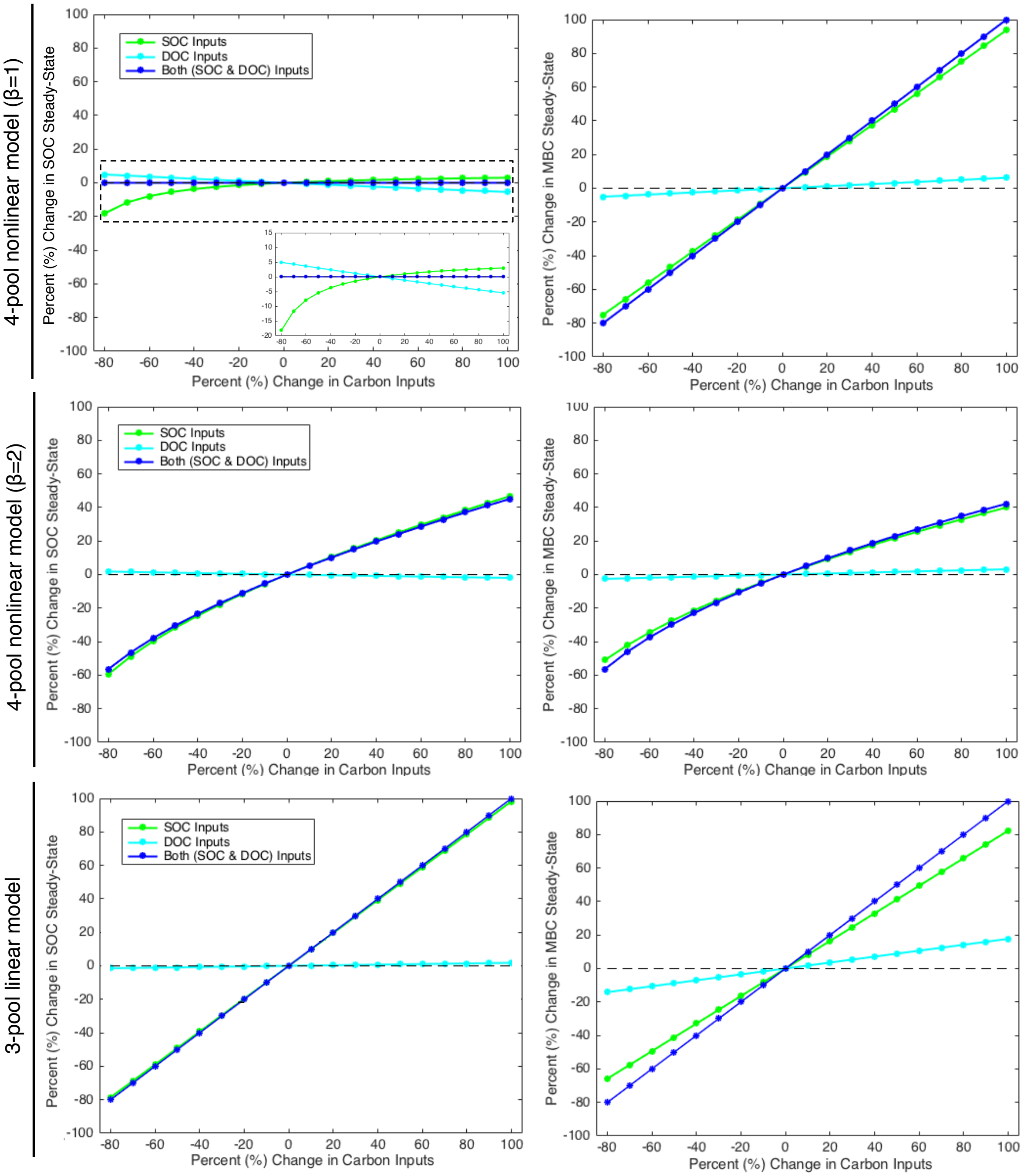

<u>Supplementary Figure 7:</u> Response of the 4-pool microbial model with and without density-dependence and the linear 3-pool model to perturbations in inputs. SOC, DOC, or both inputs are individually perturbed in each model. *Left panels:* Percent change in the SOC steady-state as a function of the percent change in C inputs, where the inset plot zooms into the dashed box. *Right panels:* Percent change in the MBC steady-state as a function of the percent change in C inputs.

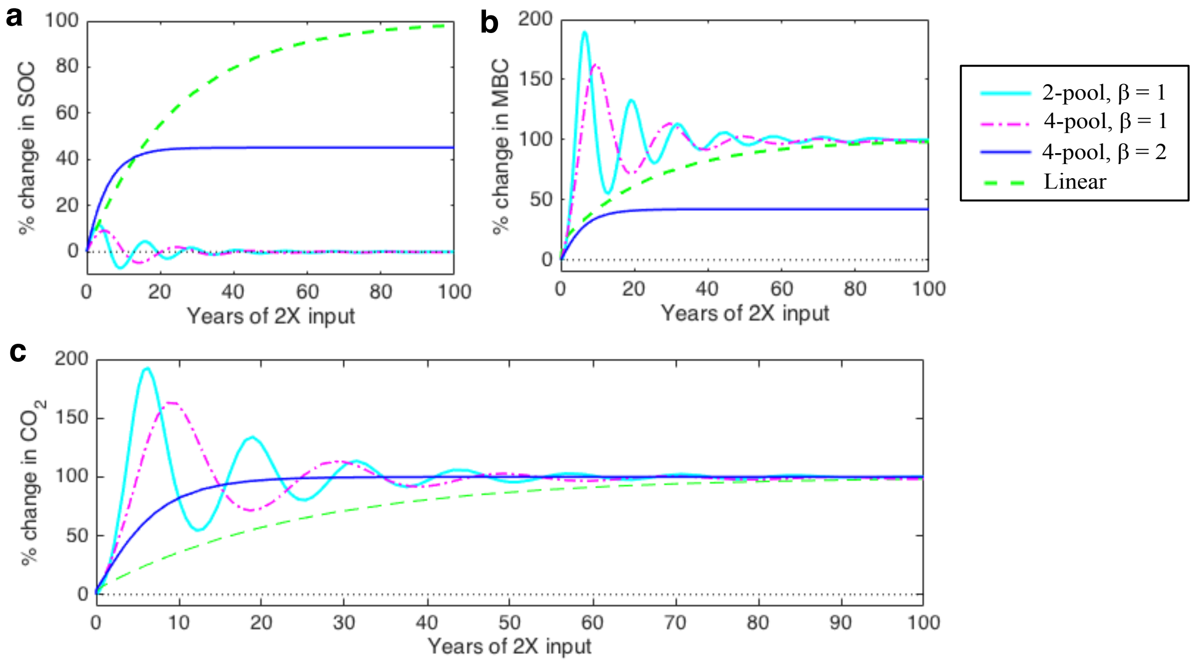

<u>Supplementary Figure 8:</u> Response of soil to doubling of plant C inputs in models. Percent change of modeled **(a)** SOC, **(b)** MBC, and **(c)** CO_2 following a doubled (2X) step increase in inputs. A value of $\beta > 1$ corresponds to a microbial model with density-dependent microbial turnover.

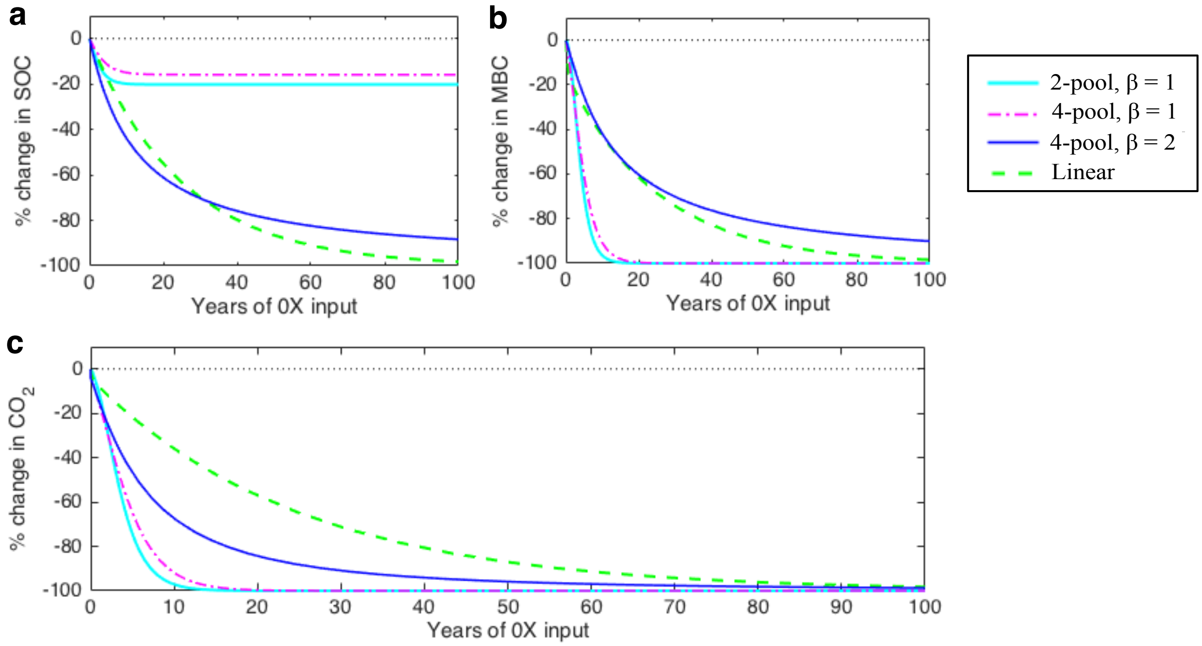

<u>Supplementary Figure 9:</u> Response of soil to removal of plant C inputs in models. Percent change of modeled **(a)** SOC, **(b)** MBC, and **(c)** CO_2 following sustained 0X inputs. A value of $\beta > 1$ corresponds to a microbial model with density-dependent microbial turnover.

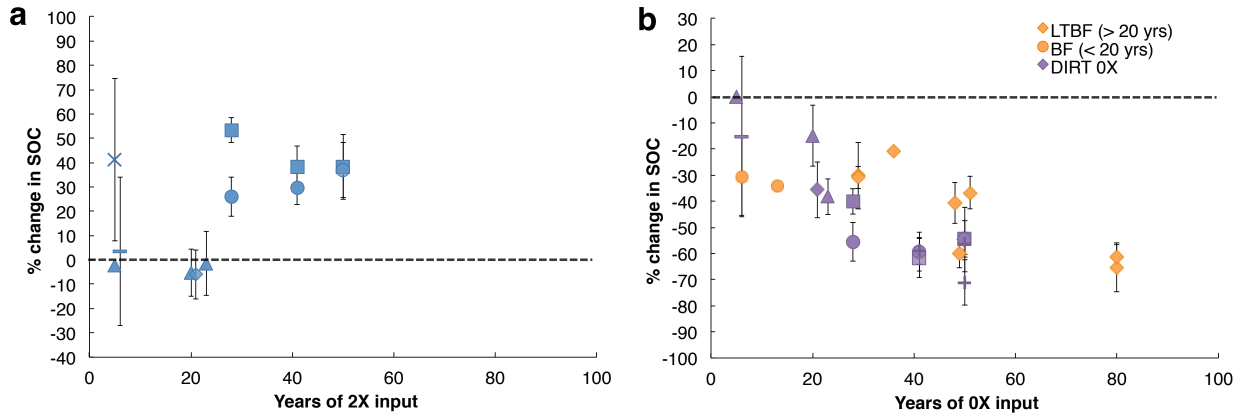

<u>Supplementary Figure 10:</u> Response of SOC to doubling and removal of plant C inputs from experiments labeled by site. (a) Percent change in SOC at Detritus Input and Removal Treatment (DIRT) experiments after a sustained 2X step increase in inputs. **(b)** Percent change in SOC at DIRT, Bare Fallow (BF) and Long-Term Bare Fallow (LTBF) experiments after sustained 0X inputs. Points indicate means and bars the standard error of the mean. Data sources are reported in Supplementary Tables 2-3. For the DIRT 2X and 0X experiments, the sites are depicted by the following marker styles in blue and purple, respectively: squares = Noe woods, circles = Wingra woods, + = Curtis prairie, triangles = Harvard, diamond = Bousson, dash = Sikfokut, x = HJ Andrews.

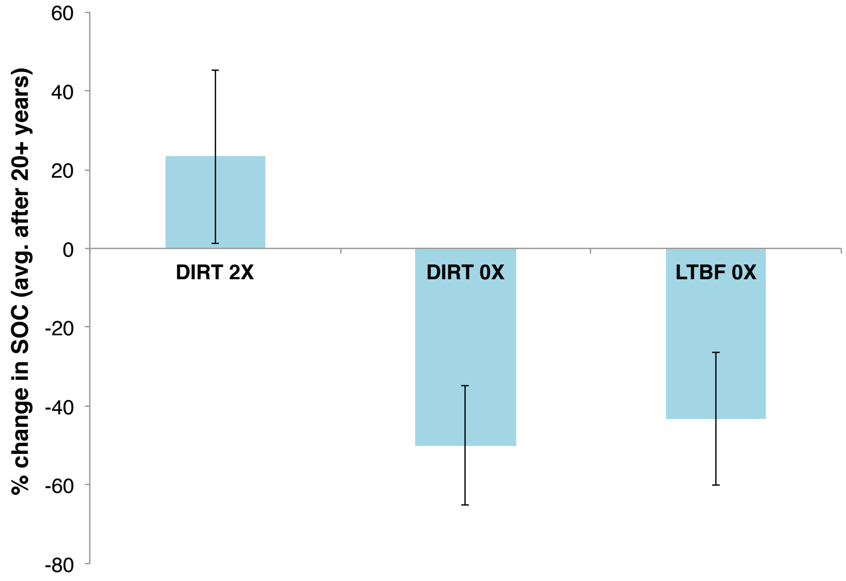

<u>Supplementary Figure 11:</u> Response of SOC to doubling and removal of plant C inputs from experiments. Average percent change in SOC after 20+ years of litter manipulation across all Detritus Input and Removal Treatment (DIRT) and Long-term Bare Fallow (LTBF) sites, which consistently doubled (2X) and removed (0X) inputs to the soil over time. Points indicate means and bars the standard error of the mean. Data sources are reported in Supplementary Tables 2-3.

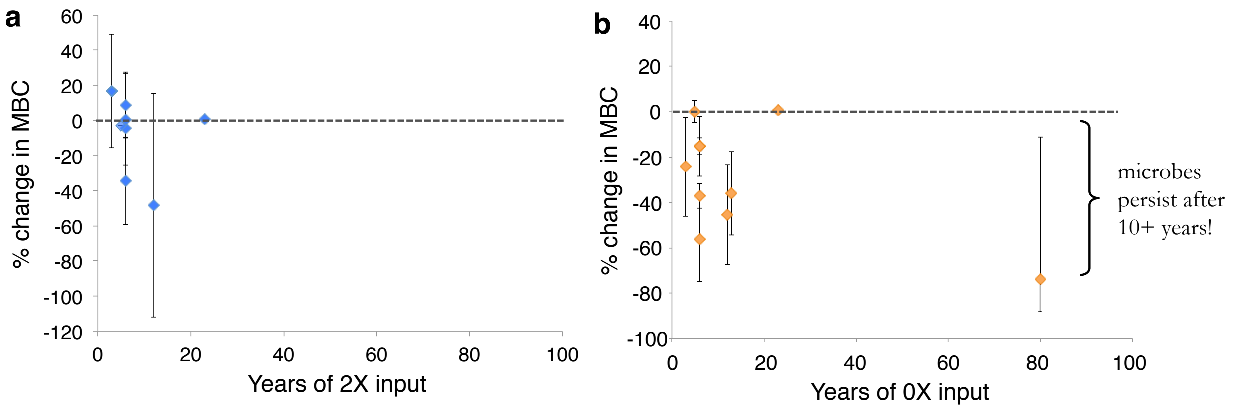

<u>Supplementary Figure 12:</u> Response of microbial biomass carbon to doubling and removal of plant C inputs from experiments. (a) Percent change of microbial biomass carbon (MBC) at DIRT experiments after a sustained doubling (2X) in inputs. **(b)** Percent change of MBC at DIRT, BF and LTBF experiments after sustained removal (0X) of inputs. Points indicate means and bars the standard error of the mean. Data sources are reported in Supplementary Tables 2-3. Substantial seasonal variability was observed in ref [3]. MBC does not double in response to 2X inputs and does not disappear within 10 years of 0X, as predicted by common microbial model formulations.

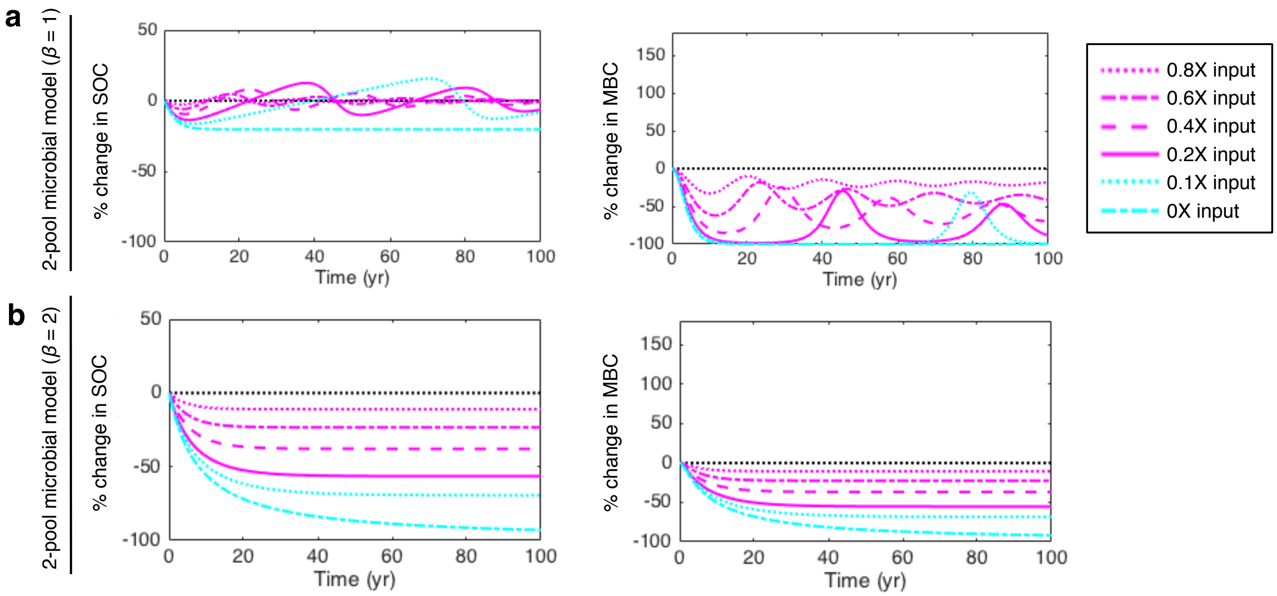

<u>Supplementary Figure 13:</u> Stability and sensitivity of the 2-pool microbial model with and without density-dependence to a decrease and complete removal of plant C inputs. (a) Percent change of modeled SOC and MBC in the 2-pool microbial model without density dependence ($\beta = 1$) following a step decrease (< 1X) and complete removal (0X) in C inputs. **(b)** Percent change of modeled SOC and MBC in the 2-pool microbial model with density-dependence ($\beta = 2$) following a step decrease (< 1X) and complete removal (0X) in C inputs.

Supplementary Table 1: Parameter values for each SOC model. Parameter values are given at a reference temperature of 20°C.

Parameter	Description	Unit	Value	Reference
**2-pool microbial model (C_S, C_B)**				
I	Plant carbon input rate	mg C g^{-1} soil hr^{-1}	0.00016	ref. [4]
$V_{max,U}$	Maximum assimilation rate	mg C mg^{-1} MBC hr^{-1}	0.01	"
$K_{M,U}$	Half-saturation for assimilation	mg C g^{-1} soil	250	"
k_B	Mortality rate	mg C mg^{-1} C hr^{-1}	0.00028	"
ε	Carbon use efficiency	-	0.31	ref. [4,5]
β	Density-dependent exponent	-	[1 to 2]	This study
**4-pool microbial model (C_S, C_D, C_B, C_E) ***				
V_{max}	Maximum decomposition rate	mg C mg^{-1} C hr^{-1}	1	ref. [4]
K_M	Half-saturation for decomposition	mg C g^{-1} soil	250	"
$V_{max,U}$	Maximum assimilation rate	mg C mg^{-1} MBC hr^{-1}	0.01	"
$K_{M,U}$	Half-saturation for assimilation	mg C g^{-1} soil	0.26	"
f	Fraction of inputs into C_S	-	0.94	"
a_{BS}	Fraction of microbial turnover into C_S	-	0.5	"
r_E	Enzyme turnover rate	mg C mg^{-1} C hr^{-1}	0.001	"
r_p	Enzyme production rate	mg C mg^{-1} MBC hr^{-1}	5.6×10^{-6}	"
β	Density-dependent exponent	-	[1 to 2]	This study
**5-pool microbial model (C_S, C_D, C_B, C_E, C_q) ***				
k_{ads}	Adsorption rate constant	mg C mg^{-1} C hr^{-1}	0.01	This study; ref [6]
k_{des}	Desorption rate constant	mg C mg^{-1} C hr^{-1}	0.001	This study; ref [6]
Q_{max}	Maximum DOC adsorption capacity	mg C g^{-1} soil	1.7	ref. [7]
**3-pool linear model (C_S, C_D, C_B)**				
k_S	Decomposition rate constant of SOC	mg C mg^{-1} C hr^{-1}	5.6×10^{-6}	ref. [4]
k_D	Decomposition rate constant of DOC	mg C mg^{-1} C hr^{-1}	0.001	"
k_B	Turnover rate constant of MBC	mg C mg^{-1} C hr^{-1}	0.00028	"
k_{uptake}	Uptake rate constant of DOC	mg C g^{-1} DOC hr^{-1}	0.0005	"
f_S	Fraction of SOC entering DOC	-	0.31	"
f_D	Fraction of DOC entering SOC	-	0.31	"
f_B	Fraction of MBC turnover recycled	-	0.31	"
$f_{B\to S}$	Fraction of recycled MBC into SOC	-	0.5	"

* Unless otherwise noted, as complexity is added in subsequent microbial models, all parameters that have an analogous value in a simpler model conserve their value in the more complex models.

<u>Supplementary Table 2:</u> Summary of Detritus Input and Removal Treatment experiments synthesized in our study. Data sources and details for the synthesized Detritus Input and Removal Treatment (DIRT) experiments are listed.

Source	Location	Type	Duration (yrs)	SOC	MBC
**DIRT (>20 years)**					
Lajtha et al. 2014 (ref. [8])	Noe	2X	50	y	-
	Noe	2X	41	y	-
	Noe	2X	28	y	-
	Wingra	2X	50	y	-
	Wingra	2X	41	y	-
	Wingra	2X	28	y	-
	Noe	0X	50	y	-
	Noe	0X	41	y	-
	Noe	0X	28	y	-
	Wingra	0X	50	y	-
	Wingra	0X	41	y	-
	Wingra	0X	28	y	-
	Curtis 1	0X	50	y	-
	Curtis 1	0X	41	y	-
	Curtis 3	0X	50	y	-
Rousk & Frey 2015 (ref. [9])	Harvard	2X	23	y	y
	Harvard	0X	23	y	y
Lajtha et al. 2014 (ref. [10])	Harvard	2X	20	y	-
	Harvard	0X	20	y	-
Bowden et al. 2014 (ref. [11])	Bousson	2X	21	y	-
	Bousson	0X	21	y	-
**DIRT (<20 years)**					
Crow et al. 2009 (ref. [12])	HJ Andrews	2X	5	y	-
Brant et al. 2006 (ref. [13])	HJ Andrews	2X	6	-	y
	HJ Andrews	0X	6	-	y
Brant et al. 2006 (ref. [3])	Bousson	2X	12	-	y
	Bousson	0X	12	-	y
	HJ Andrews	2X	6	-	y
	HJ Andrews	0X	6	-	y
	HJ Andrews	2X	6	-	y
	HJ Andrews	0X	6	-	y
	HJ Andrews	2X	6	-	y
	HJ Andrews	0X	6	-	y
	Sikfokut	2X	3	-	y
	Sikfokut	0X	3	-	y
Nadelhoffer et al. 2004 (ref. [14])	Harvard	2X	5	y	y
	Harvard	0X	5	y	y
Fekete et al. 2011 (ref. [15,16])	Sikfokut	2X	6	y	-
	Sikfokut	0X	6	y	-

<u>Supplementary Table 3:</u> **Summary of Long-term Bare Fallow and Bare Fallow experiments synthesized in our study.** Data sources and details for the synthesized Long-term Bare Fallow (LTBF) and Bare Fallow (BF) experiments are listed.

Source	Location	Type	Duration (yrs)	SOC	MBC
**LTBF (>20 years)**					
Barre et al. 2010 (ref. [17])	Kursk	0X	36	y	-
	Ultuna	0X	51	y	-
	Askov B3	0X	29	y	-
	Askov B4	0X	29	y	-
	Grignon	0X	48	y	-
	Versailles	0X	80	y	-
	Rothamsted	0X	49	y	-
Guenet et al. 2011 (ref. [18])	Versailles	0X	80	y	y
**BF (<20 years)**					
Pothoff et al. 2006 (ref. [19])	UC Hastings	0X	6	y	y
Wang et al. 2007 (ref. [20])	China	0X	13	y	y

Supplementary References

1. Xu, X., Thornton, P. E. & Post, W. M. A global analysis of soil microbial biomass carbon, nitrogen and phosphorus in terrestrial ecosystems. *Glob. Ecol. Biogeogr.* **22,** 737–749 (2013).
2. Serna-Chavez, H. M., Fierer, N. & Van Bodegom, P. M. Global drivers and patterns of microbial abundance in soil. *Glob. Ecol. Biogeogr.* **22,** 1162–1172 (2013).
3. Brant, J. B., Sulzman, E. W. & Myrold, D. D. Microbial community utilization of added carbon substrates in response to long-term carbon input manipulation. *Soil Biol. Biochem.* **38,** 2219–2232 (2006).
4. Li, J., Wang, G., Allison, S. D., Mayes, M. A. & Luo, Y. Soil carbon sensitivity to temperature and carbon use efficiency compared across microbial-ecosystem models of varying complexity. *Biogeochemistry* **119,** 67–84 (2014).
5. Sinsabaugh, R. L. *et al.* Stoichiometry of microbial carbon use efficiency in soils. *Ecol. Monogr.* **86,** 172–189 (2016).
6. Ahrens, B., Braakhekke, M. C., Guggenberger, G., Schrumpf, M. & Reichstein, M. Contribution of sorption, DOC transport and microbial interactions to the ^{14}C age of a soil organic carbon profile: Insights from a calibrated process model. *Soil Biol. Biochem.* **88,** 390–402 (2015).
7. Mayes, M. A., Heal, K. R., Brandt, C. C., Phillips, J. R. & Jardine, P. M. Relation between Soil Order and Sorption of Dissolved Organic Carbon in Temperate Subsoils. *Soil Sci. Soc. Am. J.* **76,** 1027–1037 (2012).
8. Lajtha, K. *et al.* Changes to particulate versus mineral-associated soil carbon after 50 years of litter manipulation in forest and prairie experimental ecosystems. *Biogeochemistry* **119,** 341–360 (2014).
9. Rousk, J. & Frey, S. D. Revisiting the hypothesis that fungal-to-bacterial dominance characterises turnover of soil organic matter and nutrients. *Ecology* **85,** 457–472 (2015).
10. Lajtha, K., Bowden, R. D. & Nadelhoffer, K. Litter and Root Manipulations Provide Insights into Soil Organic Matter Dynamics and Stability. *Soil Sci. Soc. Am. J.* **78,** S261 (2014).
11. Bowden, R. D. *et al.* Litter Input Controls on Soil Carbon in a Temperate Deciduous Forest. *North Am. For. Soils* S66–S75 (2014). doi:10.2136/sssaj2013.09.0413nafsc
12. Crow, S. E. *et al.* Increased coniferous needle inputs accelerate decomposition of soil carbon in an old-growth forest. *For. Ecol. Manage.* **258,** 2224–2232 (2009).
13. Brant, J. B., Myrold, D. D. & Sulzman, E. W. Root controls on soil microbial community structure in forest soils. *Oecologia* **148,** 650–659 (2006).
14. Nadelhoffer, K. J. *et al.* The DIRT Experiment: Litter and Root Influences on Forest Soil Organic Matter Stocks and Function. *For. time Environ. consequences 1000 years Chang. New Engl.* 300–315 (2004).
15. Fekete, I., Varga, C., Kotroczó, Z., Tóth, J. A. & Várbiró, G. The relation between various detritus inputs and soil enzyme activities in a Central European deciduous forest. *Geoderma* **167–168,** 15–21 (2011).
16. Fekete, I., Kotroczó, Z., Varga, C., Veres, Z. & Tóth, J. A. The effects of detritus input on soil organic matter content and carbon dioxide emission in a central European deciduous forest. *Acta Silv. Lignaria Hungarica* **7,** 87–96 (2011).
17. Barré, P. *et al.* Quantifying and isolating stable soil organic carbon using long-term bare

fallow experiments. *Biogeosciences* **7,** 3839–3850 (2010).
18. Guenet, B. *et al.* Metabolic capacities of microorganisms from a long-term bare fallow. *Appl. Soil Ecol.* **51,** 87–93 (2011).
19. Potthoff, M. *et al.* Soil microbial community composition as affected by restoration practices in California grassland. *Soil Biol. Biochem.* **38,** 1851–1860 (2006).
20. Wang, G. H. *et al.* Biomass and catabolic diversity of microbial communities with long-term restoration, bare fallow and cropping history in Chinese Mollisols. *Plant, Soil Environ.* **53,** 177–185 (2007).
